# Aortic Aneurysm as a Complication of Granulomatosis with Polyangiitis Successfully Treated with Prednisolone and Cyclophosphamide: A Case Report and Review of the Literature

**DOI:** 10.1155/2018/9682801

**Published:** 2018-06-05

**Authors:** Naoko Niimi, Tomoko Miyashita, Kana Tanji, Takuya Hirai, Kozo Watanabe, Keigo Ikeda, Shinji Morimoto, Iwao Sekigawa

**Affiliations:** ^1^Department of Anesthesia, Juntendo University Urayasu Hospital, 2-1-1 Tomioka, Urayasu, Chiba 279-0021, Japan; ^2^Department of Internal Medicine and Rheumatology, School of Medicine, Juntendo University, 2-1-1 Hongo, Bunkyo, Tokyo 113-8421, Japan; ^3^Department of Internal Medicine and Rheumatology, Juntendo University Urayasu Hospital, 2-1-1 Tomioka, Urayasu, Chiba 279-0021, Japan

## Abstract

A 57-year-old Japanese man was admitted to the hospital with back pain and fever, multiple lung nodules, and abdominal aortic aneurysm (AAA). Laboratory tests performed at admission showed an increased proteinase 3 anti-neutrophil cytoplasmic antibody (PR3-ANCA) level. Video-associated thoracoscopic lung biopsy was performed; pathologic examination showed granulation tissue with necrosis and multinucleated giant cells. The diagnosis of granulomatosis with polyangiitis (GPA) was confirmed on the basis of the clinical presentation, laboratory findings, and lung biopsy. All symptoms were ameliorated, and the serum level of PR3-ANCA declined following treatment with prednisolone and cyclophosphamide. Although the association of GPA with AAA is rare, GPA may be included among the large vessel vasculitides that can give rise to aortic aneurysm.

## 1. Introduction

Granulomatosis with polyangiitis (GPA) is characterized by a systemic necrotizing vasculitis of medium-sized and small blood vessels [1]. Serum PR3-ANCA has a high sensitivity and specificity for the diagnosis of active GPA (>90%). GPA is characterized by granulomatous upper airway involvement in approximately 90% of patients and renal involvement with necrotizing and crescentic glomerulonephritis in 75% of patients [2]. Chronic inflammation can lead to arterial aneurysm formation, a characteristic of medium-sized vessel vasculitis but a very unusual feature of GPA. Large vessel and medium vessel aneurysms have rarely been described [1]. It is important to recognize aortic or other large vessel involvement owing to the high risk of rupture, dissection, and death.

Here, we report a patient with GPA diagnosed by video-associated thoracoscopic lung biopsies of bilateral lung nodules complicated by an aortic aneurysm and positive anti-PR3-ANCA. The patient underwent successful treatment with prednisolone and cyclophosphamide. The findings were compared with several published reports of large and medium vessel aneurysms in GPA.

## 2. Case Presentation

**Figure 1:**
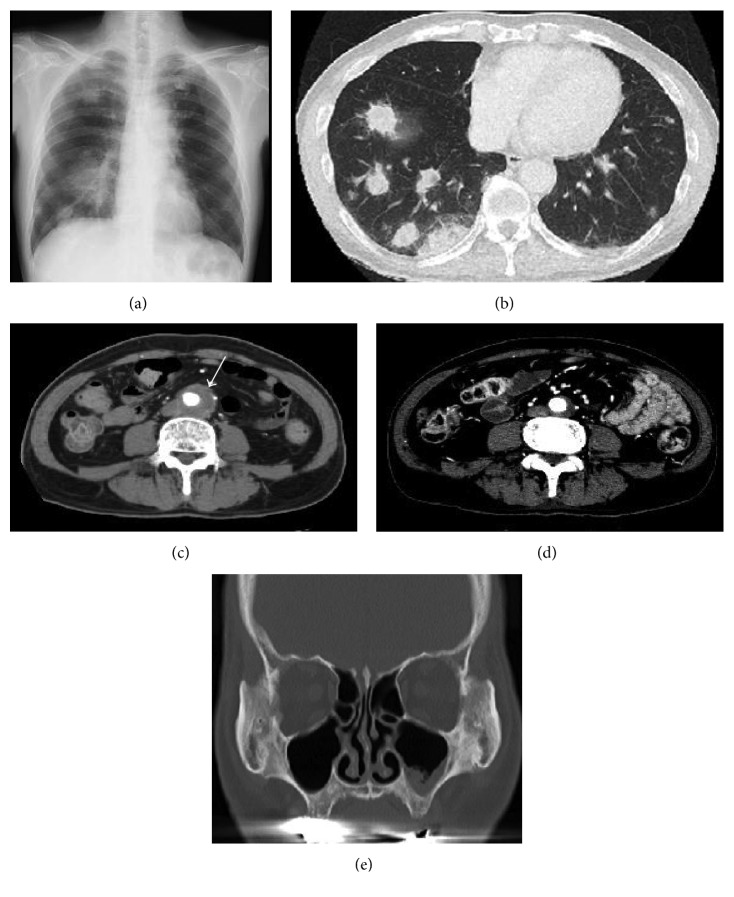
Chest radiograph and computed tomography (CT) of our patient with granulomatosis with polyangiitis. (a) Chest radiography demonstrating lung nodules. (b) CT of the chest showing lung nodules bilaterally. (c) CT of the abdomen showing a localized abdominal aortic aneurysm with a periaortic soft tissue mass. (d) CT of the abdomen after treatment. (e) CT of the left maxillary sinus.

A 57-year-old Japanese man was admitted to our hospital with a chief complaint of back pain and fever for one month. A computed tomography (CT) scan showed an aneurysm of the infrarenal aorta, with a diameter of 34 mm, and inflammation of the surrounding adipose tissue, nodular lesions of the bilateral lungs, and left maxillary sinusitis (Figure 1). He was referred to our hospital for further evaluation and treatment.

At the time of admission, the patient was 168 cm tall and weighed 56.6 kg. His blood pressure was 98/69 mmHg, pulse was 84 beats per minute, and body temperature was 39.6°C. Serum creatinine was 0.66 mg/mL, and urinalysis showed 1+ occult blood; urinary sediment contained 20 red blood cells per high-power field. Serum analysis showed leukocytosis (10,000/μL) and an elevated C-reactive protein level (29.5 mg/dL). The anti-PR3-ANCA level was 187 IU/mL, and the anti-MPO-ANCA level was normal. The abdominal aortic aneurysm was suspected to be infected, and we began administering antibiotics. The patient's general medical condition failed to improve.

**Figure 2:**
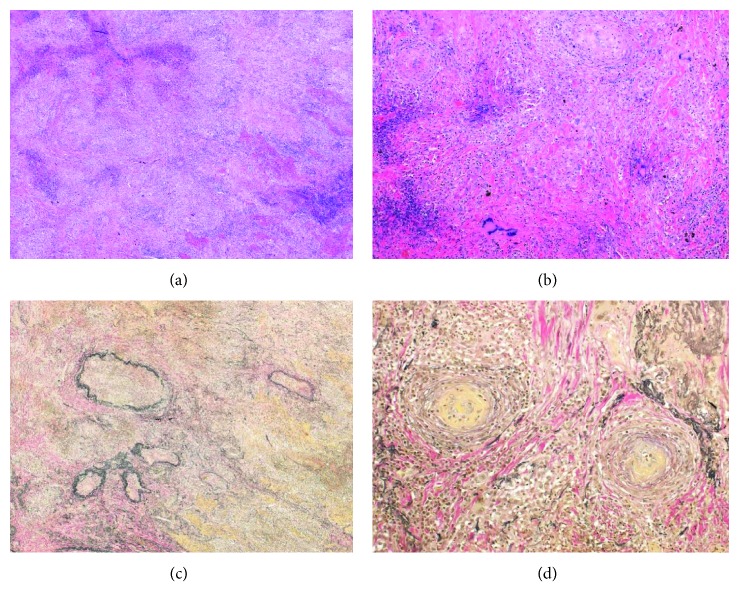
Histopathological findings of lung nodules from our patient with granulomatosis with polyangiitis. (a) Hematoxylin and eosin (H&E) staining (×40) of lung biopsy showing necrosis and inflammatory cell infiltration. (b) H&E staining (×100) of lung biopsy showing vascular occlusion and multinucleated giant cells. (c) Elastica van Gieson (EVG) staining (×40) of the lung showing destruction of the arterial medium. (d) EVG staining (×100) of lung biopsy showing vascular occlusion.

The clinical findings of left maxillary sinusitis, multiple nodular lesions in the lungs, fever, and positive anti-PR3-ANCA were clinically suspicious for GPA. Video-associated thoracoscopic lung biopsy was performed. The biopsy specimens demonstrated granulation tissue with necrosis and multinucleated giant cells (Figure 2). Most infiltrating cells were neutrophils. The ratio (%) of IgG4 to total IgG-positive cells was 33%, and there were 60 IgG4+ plasma cells per HPF in the lung.

The patient was diagnosed with GPA. He was treated with an intravenous semipulse dose of methylprednisolone, followed by oral prednisolone 1 mg/kg (55 mg) per day and intravenous administration of cyclophosphamide (700 mg/body once per month). All of the patient's symptoms and CT findings rapidly improved (Figure 1(d)), and the PR3-ANCA level promptly decreased to the normal range. The diameter of the aneurysm changed from 34 mm to 21 mm after treatment. On tapering doses of steroids, the patient is currently in remission, and the inflammation and PR3-ANCA elevation have completely resolved. There has been no disease recurrence for 4 years after initiation of therapy.

## 3. Discussion

GPA is one of the ANCA-associated small vasculitides [3]. GPA is characterized by systemic necrotizing inflammation of small- to medium-sized blood vessels and granulomatous upper airway involvement in approximately 90% of patients; renal involvement with necrotizing and crescentic glomerulonephritis occurs in 75% of patients. Other organs including the skin, joints, heart, central nervous system, and eyes can also be affected [2].

Our patient developed lung nodules and abdominal aneurysm with inflammation, an unusual manifestation of GPA. Normally, aortic lesions are more frequently seen in large vessel vasculitis, such as giant cell arteritis and Takayasu's arteritis [4]. In our case, abdominal aneurysms showed findings of chronic periaortitis (CP) which were characterized by deposition of fibroinflammatory, periaortic tissue with the retroperitoneal tissue. The aortic diameter of CP is often normal at the time of diagnosis [5]. The differential diagnosis of CP includes abdominal aortic aneurysm, syphilitic mesoarteritis [6], IgG4-related disease (IgG4-RD) [7], Takayasu's arteritis [8], temporal arteritis [9], and infectious arteritis [10]. Takayasu's and temporal arteritis may lead to vascular occlusion and tissue ischemia. Syphilitic mesoarteritis and infectious arteritis were ruled out by serological findings and negative cultures, respectively. Takayasu's arteritis and temporal arteritis were also ruled out by the absence of vascular occlusion and tissue ischemia. We considered the possibility of a case of overlapping GPA and IgG4-RD. It was unclear whether the CP was due to vasculitis, granulomatous inflammation, or a predominant IgG4-RD-like pathology because histological samples of the aorta were not available. Danlos et al. proposed an overlap of IgG4-RD/ANCA-associated vasculitides (AVV) [11]. In their study, nearly half the patients met only one of the two immunohistological criteria of IgG4-RD (IgG4+/IgG+ plasma cell ratio > 40% and >10IgG4+ plasma cell per HPF) although tissue samples were obtained from the lesion with a typical IgG4-RD organ, and histologic patterns did not overlap in the same tissue [12]. Therefore, some patients that they considered to have overlap syndrome of IgG4-RD and AVV had no histological confirmation of IgG4-RD but did have suggestive manifestations and elevated serum IgG4 levels. Our patient had slightly elevated serum IgG4 (131 mg/dL) and CP. The ratio of IgG4-positive plasma cells to IgG-positive plasma cells (33%) did not meet the criteria for IgG4-RD, but the amount of IgG4-positive plasma cells per high-power field was very high. Thus, our patient was most likely to have overlap of IgG4-RD and GPA.

Arterial aneurysm is a very rare complication of GPA. A review of the literature identified 20 cases with arterial aneurysms in GPA, including aortic involvement in 10 patients (Table 1) [1, 13–30]. Of the 20 total reported cases, in 9 patients the aneurysm ruptured, leading to death from hemorrhage in 5 patients. All of these patients were treated with medications such as steroids and cyclophosphamide, but medical treatment could not prevent aortic rupture, and the patients ultimately died. In the remaining 11 patients, including 7 with aneurysms of the aorta that did not rupture, disease remission was achieved with surgery in 6 patients and medication with prednisolone and cyclophosphamide in 1 patient. Rupture was prevented in all 10 patients who were treated surgically.

Patients with aortic aneurysm can be managed via medical or surgical approaches. Similarly, arterial aneurysms in patients with GPA can be managed by both approaches. Corticosteroids and cyclophosphamide are reportedly effective for the treatment of GPA with arterial aneurysms. Surgery is generally recommended when the diameter of an abdominal aortic aneurysm is >60 mm. Endovascular aneurysm repair (EAR) is recommended when inflammatory aortic aneurysm (IAA) causes the thickness of retroperitoneum or adhesion of urinary duct. Medication is recommended in the case of infectious aneurysm [31]. We decided to administer corticosteroids and cyclophosphamide because the diameter of the aneurysm in our patient was 34 mm; we chose not to perform select EAR because we could not rule out the possibility of infectious aneurysm. IAA is often treated with EAR, which can reduce the risk associated with severe intraoperative cardiac and pulmonary complications [32]. Immunosuppressive therapies could reduce adventitial thickening; however, we needed to consider the risk of aneurysmal rupture due to thinning of the adventitia. The condition of the aneurysm should be checked by imaging frequently because it cannot be predicted when the aneurysm might rupture after treatment.

In conclusion, we present a rare case of GPA with lung nodules and abdominal aortic aneurysm. GPA should be included in the differential diagnosis of large vessel vasculitis, which can give rise life-threatening periaortic inflammation. The possibility of aneurysmal rupture should be carefully considered when administering immunosuppressive therapies for GPA with aneurysm.

## Figures and Tables

**Table 1:** Case of aortic involvement in granulomatosis with polyangiitis.

Case	Age/gender (years)	Affected site	Antibodies	Duration of complaint	Treatment	Rupture	Outcome
1 [1]	38/M	TAA	Anti-neutrophil cytoplasmic antibodies × 128	NS	Surgery (J-graft) + PSL 15 mg/day	Yes	Good
2 [13]	51/M	Distal part of aorta (3.8 cm)	Anti-proteinase-3 antibodies > 530 kU/L	2 months	Steroid pulse + PSL 1 mg/kg + CY 2 mg/kg/day	No	Good
3 [14]	45/M	Aorta, extending to the right iliac artery	ANCA + NS	5 days	Right ureterolysis + immunosuppressive therapy	No	Good
4 [15]	33/M	AAA	Antiproteinase-3 (>1/10)	3 weeks	IJV graft + PSL + CY	No	Good
5 [16]	42/M	AAA	Antiproteinase-3 157 AU/l	1 month	Aortoiliac graft and high-dose PSL + CY 2 mg/kg/day	No	Good
6 [17]	63/M	AAA (5.4 cm)	p-ANCA 1/80 and MPO 28 U/l	2 months	Surgery + PSL 1 mg/kg + CY 2 mg/kg	No	Good
7 [18]	50/F	TAA	p-ANCA (1 : 320) antimyeloperoxidase 440 U/ml	2 months	PSL + CY	Yes	Death
8 [19]	43/M	Infrarenal aorta (3.1 × 3.5 cm)	NS	1 week	PSL + surgery	No	Good
9 [20]	29/M	Branches of hepatic and renal arteries	Anti-PR3 ANCA 15 IU/mL	NS	Coil embolization + steroid pulse + PSL 60 mg + MMF 2.5 g	No	Good
10 [21]	34/M	Anterior choroidal artery	PR3 ANCA 457 EU	1 year	Clipping + steroid pulse + PSL 40 mg + CY	Yes	Good
11 [22]	67/M	Superior pancreaticoduodenal artery	C-ANCA 1 : 512 PR3 ANCA 88 IU/L	11 months	IVCY (0.7 g/m2) every 3 weeks + steroid pulse + PSL 1 mg/kg/day	Yes	Death
12 [23]	58/F	Subclavian aneurysm	P-ANCA 68 units	2 months	PSL 1 mg/kg + CY 2 mg/kg + stent graft	No	Good
13 [24]	56/M	Left gastric artery	C-ANCA positive	1 month	None	Yes	Death
14 [25]	55/M	Hepatic artery	C-ANCA 1 : 80	3 weeks	mPSL + CY	Yes	Death
15 [26]	24/M	Bilateral renal artery	NS	6 weeks	PSL 30 mg + CY 150 mg/day	Yes	Good
16 [27]	59/M	Aorta	C-ANCA 158 SLI units	9 months	Coronary artery bypass + steroid pulse + PSL + CY 3 mg/kg + plasmapheresis	No	Good
17 [28]	35/M	Hepatic, renal, splanchnic	C-ANCA positive	6 weeks	Steroid pulse + PSL + IVCY 750 mg	Yes	Good
18 [29]	30/M	Renal artery	NS	1 month	PSL 1 mg/kg/day and CY 2 mg/kg/day	No	Good
19 [30]	53/F	Renal artery	NS	20 days	PSL 1 mg/kg/day + CY 2 mg/kg/day + hemodialysis	No	Good
20 [30]	79/M	TAA	PR3-ANCA 1180 EU	8 months	Steroid pulse PSL 60 mg + IVCY 300 mg	Yes	Death
Present case	58/M	AAA	PR3 ANCA 187 IU/ml	2 weeks	Steroid pulse + PSL 55 mg + IVCY 500 mg	No	Good

TAA: thoracic aortic aneurysm; AAA: abdominal aortic aneurysm; F: female; M: male; NS: not stated; PSL: prednisolone; CY: cyclophosphamide; mPSL: methylprednisolone; MMF: mycophenolate mofetil; IVCY: intravenous cyclophosphamide; IJV: internal jugular vein.
